# Synthesis and Evaluation of a Molecularly Imprinted Polymer for Selective Solid-Phase Extraction of Irinotecan from Human Serum Samples

**DOI:** 10.3390/jfb3010131

**Published:** 2012-02-20

**Authors:** Béatrice Roy, Sung Vo Duy, Jean-Yves Puy, Charlotte Martin, Jérome Guitton, Charles Dumontet, Christian Périgaud, Isabelle Lefebvre-Tournier

**Affiliations:** 1 Institut des Biomolécules Max Mousseron, Université Montpellier 2, UMR 5247 CNRS-UM1-UM2, case courrier 1705, Place Eugène Bataillon, Montpellier Cedex 05 F-34095, France; E-Mails: beatrice.roy@univ-montp2.fr (B.R.); sung.voduy@yahoo.fr (S.V.D.); jean-yves.puy@univ-montp2.fr (J.Y.P.); charlotte.martin@univ-montp2.fr (C.M.); christian.perigaud@univ-montp2.fr (C.P.); 2 Laboratoire de Ciblage Thérapeutique en Cancérologie, Hospices Civils de Lyon, Centre Hospitalier Lyon Sud, Pierre Bénite F-69495, France; E-Mail: jerome.guitton@univ-lyon1.fr; 3 Laboratoire de Cytologie Analytique, Inserm U590, Université Lyon 1, Lyon F-69008, France; E-Mail: charles.dumontet@chu-lyon.fr

**Keywords:** molecularly imprinted polymer, irinotecan, human serum, LC-PDA

## Abstract

A molecularly imprinted polymer (MIP) was synthesized by non-covalent imprinting polymerization using irinotecan as template. Methacrylic acid and 4-vinylpyridine were selected as functional monomers. An optimized procedure coupled to LC-PDA analysis was developed for the selective solid-phase extraction of irinotecan from various organic media. A specific capacity of 0.65 µmol·g^−1^ for the MIP was determined. The high specificity of this MIP was demonstrated by studying the retention behaviour of two related compounds, camptothecin and SN-38. This support was applied for the extraction of irinotecan from human serum samples.

## 1. Introduction

**Figure 1:**
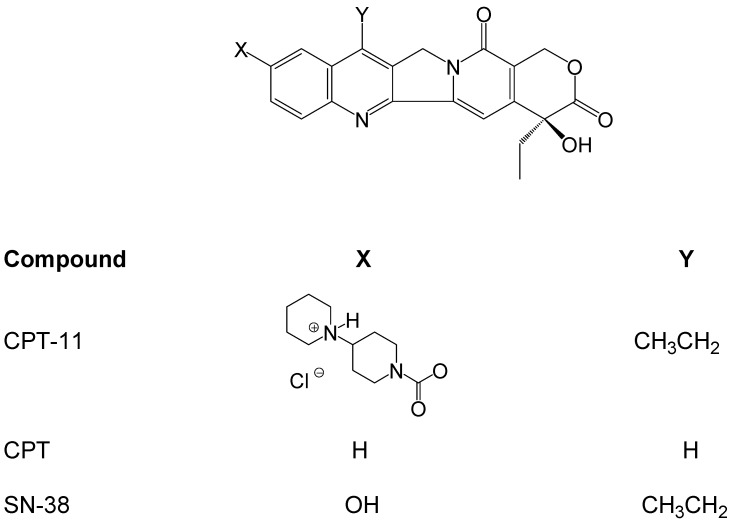
Structures of irinotecan (CPT-11), camptothecin (CPT) and SN-38.

**Figure 2:**
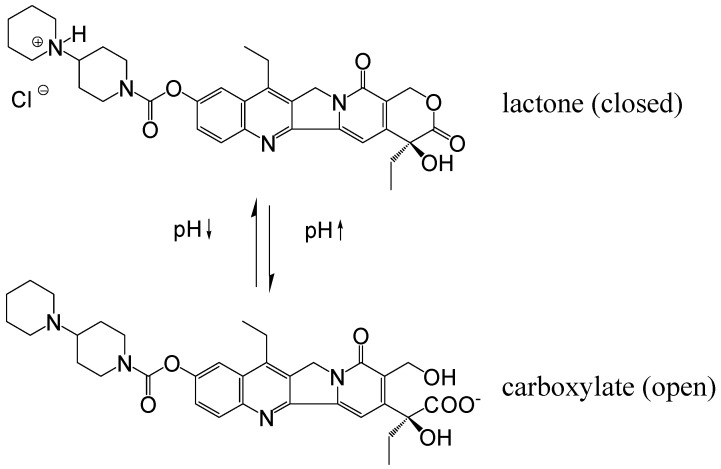
pH-dependent equilibrium between closed (active) and open (inactive) forms of irinotecan.

Irinotecan hydrochloride (CPT-11,7-ethyl-10-[4-(1-piperidino)-1-piperidino]carbonyloxy-camptothecin, Figure 1), a semisynthetic derivative of naturally occurring camptothecin (CPT), is currently used for the treatment of colorectal and gastrointestinal cancers, small cell and non-small cell lung cancer and other malignancies [1]. The cytotoxicity of irinotecan is achieved largely through a metabolite, 7-ethyl-10-hydroxycamptothecin (SN-38, Figure 1), a potent inhibitor of topoisomerase I, which is approximately 100 to 1000-fold more cytotoxic than the parent prodrug *in vitro* [2,3]. In humans, the metabolic conversion of irinotecan to the active SN-38 occurs via endogenous carboxylesterase-mediated cleavage of the carbamate bond between the camptothecin moiety and a dipiperidino side chain [4,5,6]. SN-38 is then converted to the inactive derivative SN-38 glucuronide (SN-38G) [7,8]. The α-hydroxy-δ-lactone ring of irinotecan, like all camptothecin derivatives, can undergo reversible, pH-dependent equilibration with the corresponding open chain carboxylate (Figure 2). The equilibrium favors the poorly soluble lactone form at lower pH values, whereas at neutral and basic pH values, the more soluble carboxylate form predominates [9,10]. Both forms exist *in vivo*, however only the lactone form inhibits topoisomerase and tumor growth, making this equilibrium of importance [11]. Additionally, the SN-38 lactone and carboxylate possess different affinities for transporters and exhibit differences in pharmacokinetics in animals and humans.

Several high-performance liquid chromatography methods, which have been recently reviewed by R. Mullangi *et al.* [12], allow the determination of irinotecan and its metabolites in biological fluids. Since the plasma concentrations of CPT-11 and SN-38 in lactone form correlate with the level of the total forms (lactone + carboxylate) of these two compounds [13], most pharmacokinetic studies determine the total amount of the two compounds by treating the plasma samples with low pH and then determining the lactone levels by HPLC*.* Moreover, sample processing of biological samples, such as precipitation with acidified methanol or acetonitrile, is required before the chromatographic analysis of irinotecan [12]. Three solid-phase extraction (SPE) procedures have also been described [14,15,16].

The application of molecularly imprinted polymer technology to solid-phase extraction (MISPE) has been used for both biological and environmental sample analyses [17,18,19,20]. MISPE allows the analyte of interest to be pre-concentrated while simultaneously removing interfering compounds from the matrix so that selective enrichment and clean-up are obtained, resulting in a higher accuracy and a lower detection limit in the subsequent analysis. The applicability of this method has been demonstrated with a number of compounds such as herbicides and drugs, which can be selectively extracted from samples such as blood serum and urine. Imprinting with a molecule that is non-covalently bound to the polymer creates sites of molecular recognition within the polymer structure that is based on the shape of the imprinting molecule as well as the characteristics of the functional group interactions between the template and the monomers (such as ionic bonding, H-bonding, dipolar bonding, and van der Waals interactions). The main advantages of MIPs over conventional sorbents used for sample pre-treatment are the high selectivity and affinity for the target analyte.

The aim of this work was to synthesize an MIP targeted for irinotecan applied to the treatment of complex matrices. A first characterization of the MIP was achieved in different solvents in order to demonstrate the presence of selective cavities in the MIP and to measure its specific capacity. The selectivity of the developed procedure was assessed by studying the retention of the target analyte on the non-imprinted polymer (NIP). The specificity was evaluated by comparing irinotecan retention with the retention of different analogue compounds, namely SN-38 and camptothecin. Human serum samples spiked with irinotecan were also analyzed by the MIP methodology developed in this work.

## 2. Experimental Section

### 2.1. Chemicals and Standards

Irinotecan hydrochloride trihydrate was obtained from Heraeus GmbH Pharma. SN-38 was kindly provided by Pfizer. Camptothecin (CPT) was purchased from TCI Europe. Human serum was provided by Centre Régional de Transfusion Sanguine (CRTS, Montpellier, France). HPLC-grade acetonitrile (ACN), methanol (MeOH), acetic acid (AcOH) and dichloromethane (DCM) were purchased from Carlo Erba Reactifs-SDS (Val De Reuil, France). High purity water was obtained from a Milli-Q purification system (Millipore, Saint-Quentin en Yvelines, France). SPE cartridges (1 mL) and frits were purchased from Interchim (Montluçon, France). Methacrylic acid (MAA), 4-vinylpyridine (4-VP), ethylene glycol dimethacrylate (EGDMA), 2,2'-azobis(2-methylproprionitrile) (AIBN), orthophosphoric acid, dichloroethane, acetic acid and formic acid were purchased from Sigma-Aldrich (France). Dichloroethane was distilled over P_2_O_5_. MAA was distilled under reduced pressure. EGDMA was washed consecutively with 10% NaOH, water and brine, and then dried over magnesium sulfate (MgSO_4_), filtered, and distilled under reduced pressure. AIBN was recrystallised with dried methanol. Stock solutions of CPT-11, SN-38 and CPT (at 1 mg·mL^−1^ each) were prepared by dissolving the appropriate amount of compound in DMSO. These solutions were stored at −20 °C. Working solutions were prepared extemporaneously by serial dilutions in the appropriate solvent (acetonitrile, dichloromethane, water) from the stock solutions. The stock solutions at 50 mM are 95% stable for at least 2 months at −20 °C [10].

### 2.2. HPLC Analysis

The HPLC system consisted of a Waters 2695 system (Waters, Saint-Quentin en Yvelines, France) including an autosampler, a solvent delivery system and a diode-array detector (Waters 2969). Data acquisition and analysis were conducted using Empower Pro (Waters, Milford, MA, USA). The analytical column was a C18 XBridge-Waters^®^ (3.5 µm, 100 mm × 2.1 mm i.d.). The mobile phase comprised acidified water (adjusted to pH ~3 with orthophosphoric acid) as solvent A and acetonitrile as solvent B. The presence of acid in the mobile phase prevents hydrolysis of the lactone form of the analytes to the corresponding open-ring carboxylate form. Mobile phase A was filtered before use and solvents A and B were vacuum degassed *in situ* during chromatography. A gradient from 100% solvent A to 100% solvent B was run over 12 min and then held for 6 min. The flow rate was 0.3 mL·min^−1^ and the column oven temperature was maintained at 30 °C. The photodiode-array detector was set to record signals at 254 nm (CPT, CPT-11 and SN-38) in agreement with the maximum ultraviolet absorbance wavelength [21]. The retention times for CPT-11, CPT and SN-38 under those conditions were 9.4, 10.7 and 10.9 min, respectively. 

### 2.3. Synthesis of Polymers

The estimated pKa value for the piperidino group is 9.3 [3]. To circumvent the poor solubility of irinotecan hydrochloride in organic media, a deprotonation step was performed prior to MIP synthesis. It consisted of a liquid-liquid extraction with dichloromethane (20 mL) and a saturated aqueous solution of sodium hydrogen carbonate (8 mL). The aqueous phase was extracted twice with 2 × 8 mL dichloromethane. The combined organic layers were washed with deionized water and dried with anhydrous Na_2_SO_4_. Yield of liquid-liquid extraction was 92%. The ^1^H NMR spectrum of deprotonated irinotecan was in agreement with the closed form of irinotecan. Irinotecan (template, 293 mg, 0.5 mmol), MAA (86 mg, 1 mmol), EGDMA (1.98 g, 10 mmol), 4-vinylpyridine (106 mg, 1 mmol) and AIBN (30 mg) were added to 2.8 mL of dichloroethane and the solution was transferred to a glass tube. The polymerization mixture was placed in an ice bath and was degassed with nitrogen for 10 min. The tube was sealed and heated in an oil bath maintained at 60 °C. After 24 h of polymerization, the tube was crushed and the polymer was then ground with a ball mill. Particles were sieved using a combination of two test sieves of 36 µm and 25 µm (Linker-Industrie-Technik). The 25–36 µm fraction was collected and the fine particles were removed by sedimentation in a mixture of MeOH/water (80:20 v/v). A non-imprinted polymer (NIP) was also prepared using the same procedure employed for the MIP synthesis, but in the absence of the template. 

### 2.4. MISPE Conditions

MIP and NIP columns were prepared by packing 50 mg of the respective polymers into cartridges (1 mL). The material in the cartridge was secured by two polyethylene frits. The template was removed from the MIP by washing the polymer with a MeOH/AcOH mixture (95:5 v/v) until no template was detected by LC-PDA. The synthesized material was tested by percolating different organic solvents (ACN, DCM) and hydro-organic mixtures of water/ACN spiked with 2.5 µg irinotecan hydrochloride (CPT-11). Before each use, the sorbent was conditioned with a few milliliters of the same solvent. Samples spiked with irinotecan (5 µg·mL^−1^) were prepared from stock solutions by dilution in the various percolated solvents. According to the nature of the percolated solvents, different washing solutions were used. These conditions of percolation, washing and elution are summarized in Table 1. Each fraction was then collected, concentrated up to dryness under a stream of nitrogen and dissolved in 200 µL ACN containing 0.1% acetic acid. The reconstituted sample was then injected (20 µL) onto the chromatographic system.

**Table 1:** Extraction recoveries for irinotecan under different conditions.

	Recovery (%)					
	Condition A		Condition B		Condition C	
	MIP	NIP	MIP	NIP	MIP	NIP
Load	0	10.1	0	0	0	0
Wash	0	89.2	0	26.4	0	94.0
Elute	99.8	0.6	107.7	83.5	100.3	5.8

Condition A: conditioning with 3 × 1 mL DCM. Loading 0.5 mL of irinotecan in DCM (5 µg·mL^−1^), washing with 1.5 mL DCM/MeOH (98:2 v/v) then eluting with 3 mL MeOH/AcOH (95:5 v/v).

Condition B: conditioning with 3 × 1 mL ACN. Loading 0.5 mL of irinotecan in ACN (5 mg·mL^−1^), washing first with 1 mL ACN/MeOH (98:2 v/v) then washing with 1 mL ACN/MeOH (95:5 v/v) and eluting with 3 mL MeOH/AcOH (95:5 v/v).

Condition C: conditioning with 3 × 1 mL ACN. Loading 0.5 mL of irinotecan in ACN (5 µg·mL^−1^), washing with 1.5 mL ACN/MeOH (90:10 v/v) and eluting with 3 mL MeOH/AcOH (95:5 v/v).

### 2.5. MIP Loading Capacity

The determination of the MIP capacity was performed by measuring the extraction recoveries on the MIP and on the NIP after the percolation of dichloromethane samples (0.5 mL) spiked with various amounts of irinotecan hydrochloride (0.5 to 90 µg). After a washing step in 1.5 mL mixed DCM/MeOH (98:2 v/v), irinotecan was eluted from the MIP and the NIP with 3 mL of a MeOH/AcOH mixture (95:5 v/v). Eluted fractions were collected, concentrated up to dryness under a nitrogen stream and reconstituted in 200 µL ACN containing 0.1% acetic acid. The samples were then transferred to an autosampler vial and a total volume of 20 µL was injected into the LC-PDA system.

### 2.6. Study of Cross-Reactivity

In order to probe the cross-selectivity of the MIP for structures closely related to the template molecule, we investigated the behaviour of SN-38 and camptothecin towards the polymers. MIP or NIP cartridges were conditioned using 3 mL DCM. Samples of dichloromethane (0.5 mL) spiked with each compound (5 µg·mL^−1^) were loaded onto the cartridges. Then 1.5 mL of washing solvent (DCM/MeOH, 98:2 v/v) was percolated through the polymer cartridges and compounds were further eluted with 3 mL of a MeOH/AcOH mixture (95:5 v/v). Each fraction was collected, concentrated up to dryness under a nitrogen stream and reconstituted in 200 µL acetonitrile containing 0.1% acetic acid. 20 µL of each sample were analyzed by HPLC.

### 2.7. Extraction of Irinotecan from Human Serum Samples

Human serum was filtered through a Millex-GV filter (0.22 µm, 13 mm, Millipore). In a 1.5 mL centrifuge tube, an aliquot of 700 µL human serum was spiked with 100 µL irinotecan hydrochloride in MeOH/AcOH/water 20:1:20 v/v/v (final concentration of irinotecan: 10 µg·mL^−1^) at 4 °C. After vortex-mixing, 1 mL acetonitrile was added in order to precipitate proteins. The tubes were vortex-mixed for 30 s and then centrifuged at 10,000 rpm for 10 min. The supernatant was transferred in another tube and centrifuged again under the same conditions. 1 mL of the supernatant was withdrawn and evaporated to dryness under a nitrogen stream. The dry residue was dissolved in 200 µL MeOH and then diluted with 1.8 mL ACN. 0.5 mL of this solution was percolated through the MIP. The cartridges were washed with 1.5 mL ACN/MeOH (90:10 v/v) and the analytes were eluted with 3 mL MeOH/AcOH 95:5 v/v. The eluted fractions were collected, concentrated to dryness under a nitrogen stream and then dissolved in 200 µL MeOH/AcOH/water (20:1:20 v/v/v). 40 µL of these samples were analysed by LC-PDA. A reference sample was also injected. For this purpose, 0.5 mL of the previous 2 mL ACN/MeOH (90:10 v/v) solution was taken and evaporated to dryness under a nitrogen stream. The dry residue was dissolved in 200 µL MeOH/AcOH/water (20:1:20 v/v/v) and 40 µL was injected.

## 3. Results and Discussion

### 3.1. Preparation of Molecularly Imprinted Polymer

The non-covalent approach is the most frequently used method of preparing MIPs, in which the binding sites are formed by self-assembly between the template and the monomer followed by a cross-linked co-polymerization. To perform this non-covalent MIP approach, we selected 4-vinylpyridine and methacrylic acid as monomers. Irinotecan encompasses functional groups that may form hydrogen bonds with the monomers. In order to favour hydrogen bonding between the template (*H*-*Bond* Donor: 2, *H*-*Bond* Acceptor: 8) and the monomers, a nonprotic solvent was chosen as the porogenic solvent. Since the thermal polymerisation was performed at 60 °C, 1,2-dichloroethane was selected as the porogenic solvent due to its relative high boiling point (83.5 °C). Hydrogen bonding was expected during the polymerization step between the template and the functional monomer. To circumvent the low solubility of irinotecan hydrochloride in dichloroethane, CPT-11 was deprotonated prior to polymerization, to afford a highly organic-soluble template.

### 3.2. Characterization of the Molecularly Imprinted Polymer

Samples of DCM or ACN (0.5 mL) spiked with 5 µg·mL^−1^ irinotecan were percolated on the MIP. The same experiment was carried out in parallel on the NIP in order to evaluate non-specific interactions during the retention process. The recoveries of irinotecan obtained in each fraction are reported in Table 1. In order to achieve a selective extraction, a clean-up step was introduced prior to the elution step. In this step, the clean-up solvent suppressed the non-specific interactions without disrupting the selective interactions between the MIP and the target molecule. The washing solvents were optimized to obtain maximum recovery of the analyte on the NIP and minimal recovery on the MIP. Various ratios of mixed DCM/MeOH (condition A, Table 1) and ACN/MeOH (conditions B and C, Table 1) were tested. The best results were obtained when washing was performed with 1.5 mL of 98:2 v/v DCM/MeOH followed by elution with 3 mL 95:5 v/v MeOH/acetic acid. In condition A, irinotecan was extracted with a recovery of 99.8% on the MIP and with only 0.6% of recovery on the NIP, thus demonstrating selective recognition for the template. When dichloromethane was replaced by acetonitrile (conditions B), a more polar solvent, the washing step required increased methanol percentage. In conditions C, a washing step with 90:10 v/v ACN/MeOH was performed to disrupt almost all non-specific interactions on the NIP. A selective extraction was obtained using 1.5 mL 90:10 v/v ACN/MeOH as a washing solvent followed by elution with 3 mL 95:5 v/v MeOH/AcOH. 

An easy way to apply a MIP to aqueous media such as biological fluids consists of developing a selective procedure directly in aqueous media or in hydro-organic media. While in organic solvents imprint recognition is mainly based on interaction with polar functionalities, in aqueous media the recognition of hydrophobic parts of the molecules becomes more significant. To treat plasma sample, a preliminary precipitation step with an organic solvent (methanol, acetonitrile) is commonly carried out to remove highly abundant proteins. As a mixture of 50:50 v/v water/ACN is considered to precipitate proteins from biological media, this mixture was evaluated as a percolated solvent in our experiments. In these conditions, irinotecan was strongly retained both on the MIP and on the NIP but no significant difference was observed between MIP and NIP (data not shown). Retention in this case was probably not due to specific hydrophobic interactions. Therefore, considering the high selectivity obtained for both organic solvents, we carried out the experiments in organic media.

### 3.3. MIP Binding Capacity

**Figure 3:**
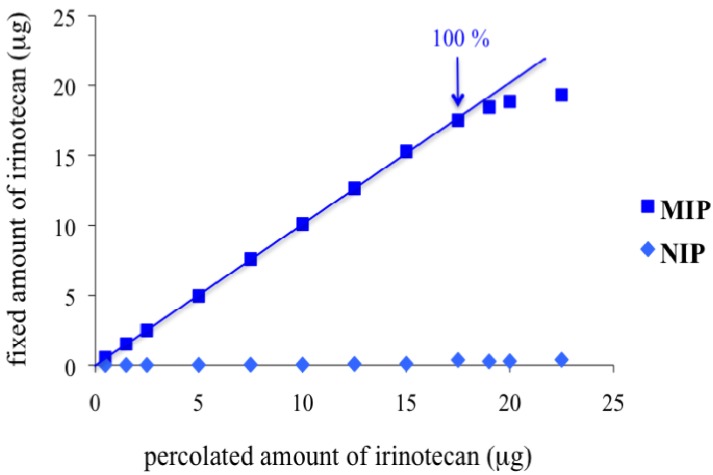
Saturation curve obtained after the percolation of 0.5 mL of dichloromethane spiked with increasing amounts of irinotecan (Condition A).

The complete characterisation of this MIP requires the measurement of its capacity, which corresponds to the maximum amount of irinotecan that can be retained on it. The determination of the capacity was performed by measuring the extraction recoveries on the MIP of samples spiked with increasing amounts of irinotecan. Experimental conditions were those described in protocol A of Table 1. Figure 3 depicts the amount of irinotecan bound on the polymers according to the amount introduced on the cartridges. The curve presents a linear part for the lowest amounts of irinotecan and tends to reach a plateau for the highest ones. As shown in Figure 3, irinotecan was completely retained on the MIP cartridge when its loaded amounts were less than 18 µg. For a percolated amount higher than 18 µg, the extraction recovery of irinotecan decreased due to saturation of the specific binding sites. The capacity was estimated to be 0.65 µmol·g^−1^ of sorbent. For percolated amounts higher than this capacity, the MIP still retains large amounts of irinotecan but with lower extraction recoveries. 

### 3.4. Retention-Behaviour of Structural Analogs on the MIP

**Figure 4:**
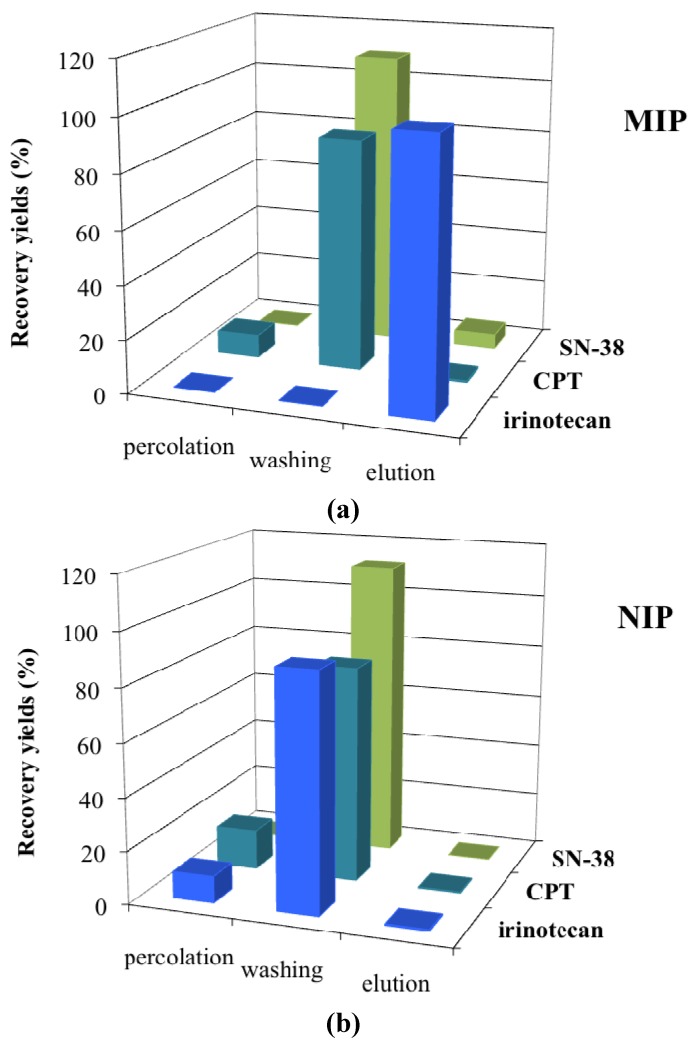
Extraction profiles obtained with (**a**) MIP and (**b**) NIP after the percolation of irinotecan (CPT-11) and its related compounds (n = 3, the RSD varied between0% and5%). Percolation: 0.5 mL DCM spiked with 2.5 µg of either irinotecan, CPT or SN-38; washing: 1.5 mL 98:2 v/v DCM/MeOH; elution: 3 mL 95:5 v/v MeOH/AcOH.

The retention of compounds structurally related to irinotecan, namely camptothecin and SN-38, was examined under condition A and compared to the retention of irinotecan. As shown in Figure 4, a low retention was obtained for CPT (0.6%) and SN-38 (5.3%) on the MIP, these compounds being removed from the MIP and the NIP during the percolation and the washing steps. The same results were observed when condition C was applied (data not shown). As depicted in Figure 1, the main structural difference between these compounds and irinotecan is the bulky piperidino moiety. Thus this group seems to be crucial for the specific recognition of irinotecan in the cavities of the polymer. These results showed that this MIP is a selective and specific support that can be used for the selective extraction of irinotecan.

### 3.5. Application of MISPE to Human Serum Samples

**Figure 5:**
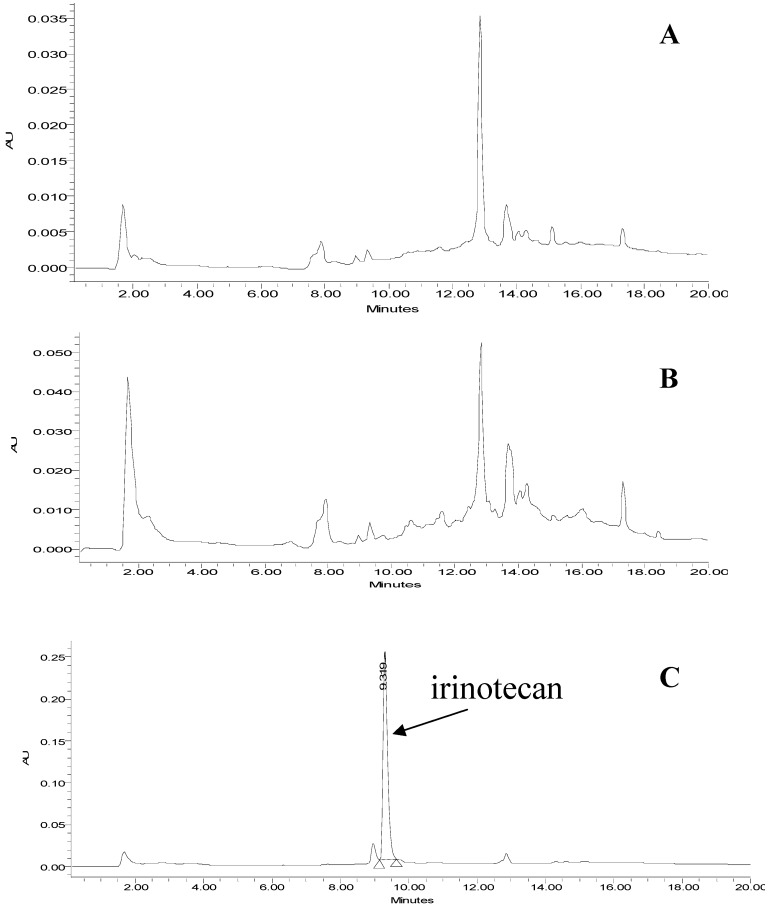
Chromatograms resulting from clean-up on MIP of human serum spiked with 10 µg·mL^−1^ of irinotecan. (**A**) Percolation fraction; (**B**) Washing fraction; (**C**) Elution fraction.

To demonstrate the potential of the polymer for the sample clean-up of complex matrices, the molecularly imprinted polymer was applied for the extraction of spiked irinotecan in human serum. Many proteins are present in human serum which may cause matrix effect during the extraction process on the MIP. For this reason, a pre-treatment step using acetonitrile was applied to our biological samples. Firstly, human serum was spiked with irinotecan in MeOH/AcOH/water (20:1:20 v/v/v) such that the final concentration of irinotecan was 10 µg·mL^−1^. Then, ACN was added allowing the quantitative precipitation of proteins. The resulting ACN/MeOH (90:10 v/v) supernatant was percolated through the polymers and processed according to condition C. Irinotecan was eluted in the washing fraction of the NIP (data not shown). Figure 5 depicts chromatograms of the collected fractions after solid phase extraction on the MIP. 

Comparison of these chromatograms demonstrates an efficient clean-up of the complex matrix. The recovery of the extraction step on MIP was determined by comparing the detector response obtained for the elution fraction with that of a non-processed sample. An extraction recovery on MIP of 88% was determined.

## 4. Conclusions

A molecularly imprinted polymer was prepared using irinotecan as template. The results presented here indicate that the polymer exhibits highly selective binding for irinotecan. A specific capacity of 0.65 µmol·g^−1^ for the MIP was determined. Study of the recognition properties of the imprinted polymer in different media was carried out. By using an optimized procedure in either acetonitrile or dichloromethane, irinotecan was extracted with a complete recovery in the eluting fractions of the MIP. An off-line protocol was developed which enabled the selective extraction of irinotecan from serum samples using the molecularly imprinted polymer as the solid-phase extraction sorbent. An extraction recovery on MIP of 88% for human serum spiked with 10 µg·mL^−1^ irinotecan was found, thus demonstrating the high selectivity brought by the MIP.
